# In Remembrance of my Mother (A Call to the Medical Community)

**DOI:** 10.1016/j.pmedr.2021.101371

**Published:** 2021-04-16

**Authors:** Corey J. Smith

**Affiliations:** Medical Director at Blue Cross and Blue Shield of Illinois, Montana, New Mexico, Oklahoma, TX, United States

It was supposed to be fun. We had planned this for years, both of us excited for what the adventure would bring. “Did you get your typhoid vaccine yet?” I asked with exhilaration, always showing preparedness instilled in me from my time as a Boy Scout. “Yes,” my mother replied with the same eagerness to match my enthusiasm. We were on our way indeed. We were going to Guangzhou, China, by way of Hong Kong. If that were not exciting enough, we were going on February 4th, 2016, which meant we would be there for Chinese New Year, the Year of the Monkey. What a celebration it would be! The lights, the fireworks, the festivities! We couldn’t wait. And the cherry on top—we would be visiting my older sister who we hadn’t seen for more than a few years. You see, my sister was residing in China, teaching English at one of the private schools just outside of Guangzhou. She invited us to experience the thrill of Chinese New Year! And we were on our way.

The only thing that separated us from our delightful destiny was a fifteen hour or so flight from San Francisco, California to Hong Kong. It was by far the longest flight either of us have taken. We were feeling all sorts of emotions, but nervousness was not one of them. How bad could the lengthy flight be? We would sleep a few hours, watch a few movies, enjoy the food and snacks on the plane, and most of all cherish each other’s company. And that is essentially what we did. We slept, my head on my mom’s shoulder at times. We enjoyed chicken and rice, ice cream, and wine, all while watching movie after movie, *The Martian* & *Black Mass* to name a few. We discussed some of the important moments in the movies we watched, and we together counted down the hours in anticipation of landing in Hong Kong. We were getting closer. We were almost there. But nothing would prepare me for what would happen upon landing, and it would change my life forever.

“We landed ok. Everything is fine,” were the last words I texted my worried younger sister who was at her home in College Park, Maryland. However, this would prove to be furthest from the truth, much to my dismay and years of subsequent grief. As we were preparing to disembark the plane and as my mom attempted to put on her shoes, she became acutely short of breath. Now, I am an MD, a medical doctor, so the differential for shortness of breath quickly raced through my worried mind. She did not have any significant medical history, so it must be anxiety, a panic attack I thought. Then as she decompensated further, I demanded the flight staff to get me supplemental oxygen, and my mind raced further—was she having a myocardial infarction, an arrhythmia, a PE, oh my, not a pulmonary embolus. Now I know how common a pulmonary embolism can be after long haul travel so I should have known the risks. “Call 911,” I screamed. She decompensated further. We moved her from the seat to the ground and started to perform CPR. The 911 ambulance came after seemingly hours. We rushed her to the hospital, our first experience in Hong Kong being raced from an airport to the hospital in an ambulance. I met my sister there. We shared tears and the pain of knowing that our beloved mother had passed away.

I write this essay not for you to feel sorry for me. Not for you to share in my grief, but as a call to all health care providers, whether they be Medical Doctors, Doctors of Osteopathy, or Nurse Practitioners, to change the way they practice. Airlines, too, are not exempt from this crusade to keep passengers safe and healthy as I feel there should be an announcement before every flight, educating passengers how to mitigate the risk of deep venous thrombosis, but I digress. I want to keep this essay focused on the health care providers, whether specialists or primary care providers. So, I write this essay to urge medical providers all across the world to have that discussion with their elderly patients before a long trip, whether a flight or a long road trip, to get up every few hours or so to walk around and stretch their legs as advocated by the American Society of Hematology (Clots and Travel, 2021). And it is not just limited to elderly patients, as other populations are at risk for deep venous thrombosis during long haul travel including those on birth control, those who have had recent surgery, those who have active malignancy, postpartum women, and those with a prior history of deep venous thrombosis (Clots and Travel, 2021). These populations would equally benefit from receiving the counseling to which I have alluded. The literature also suggests educating those at-risk patients about simple calf stretches they can do in their seats and drinking plenty of water, which both have been shown to mitigate the risk of deep venous thrombosis in patients during long haul travel (Air Travel Health Tips, 2020). The American Society of Hematology also recommends the wearing of compression stockings during lengthy travel to reduce the risk of deep venous thrombosis for those in the at-risk populations (Gavish and Brenner, 2011, "Planes). As a primary care physician myself, I am strongly debating advocating for at risk patients to take a daily aspirin starting a few days before their trip to try to minimize the chances of developing blood clots during lengthy travel if, of course, they have no contraindication to anti-platelet therapy. This suggestion is not as novel or revolutionary as it may seem, as the American Society of Hematology suggests aspirin therapy for long haul travel in those patients who have risk factors for deep venous thrombosis but are uncomfortable wearing compression stockings or taking low molecular weight heparin (Planes, Trains, and VTEs, 2019). The doses of aspirin that I have seen in literature review range from 81 mg to 160 mg, 30 min before the flight (Geroulakos, 2001, Air Travel Health Tips, 2020). Furthermore, I will take it a step further. Although low molecular weight heparin is suggested in the literature and advocated by the American Society of Hematology to protect our at risk loved ones from the potential of developing a clot during long haul travel (Planes, Trains, and VTEs, 2019), I truly believe that studies should be done on direct oral anticoagulants (DOACs) because, unlike Warfarin, these drugs have the potential to offer protection without the burden of INR checks or diet modifications. I truly believe with such pharmaceutical help, my beloved mother, Carmella Smith, would be still with us today, and we would have enjoyed that trip to China about which we were so excited.
